# Combining Propensity Scores and Common Items for Test Score Equating

**DOI:** 10.1177/01466216251363240

**Published:** 2025-07-30

**Authors:** Inga Laukaityte, Gabriel Wallin, Marie Wiberg

**Affiliations:** 1 Department of Applied Educational Science, Umeå University, Sweden; 2 School of Mathematical Sciences, Lancaster University, UK; 3 Umeå School of Business, Economics and Statistics, Department of Statistics, Umeå University, Sweden

**Keywords:** educational testing, academic admission, fairness, equating, nonequivalent groups with anchor test design

## Abstract

Ensuring that test scores are fair and comparable across different test forms and different test groups is a significant statistical challenge in educational testing. Methods to achieve score comparability, a process known as test score equating, often rely on including common test items or assuming that test taker groups are similar in key characteristics. This study explores a novel approach that combines propensity scores, based on test takers’ background covariates, with information from common items using kernel smoothing techniques for binary-scored test items. An empirical analysis using data from a high-stakes college admissions test evaluates the standard errors and differences in adjusted test scores. A simulation study examines the impact of factors such as the number of test takers, the number of common items, and the correlation between covariates and test scores on the method’s performance. The findings demonstrate that integrating propensity scores with common item information reduces standard errors and bias more effectively than using either source alone. This suggests that balancing the groups on the test-takers’ covariates enhance the fairness and accuracy of test score comparisons across different groups. The proposed method highlights the benefits of considering all the collected data to improve score comparability.

## Introduction

Assessment tests in education are important tools for measuring students’ knowledge, skills, and development. These tests also play a significant role in educational decision-making, influencing everything from teaching practices to college admissions. Given their impact, it is essential to ensure that test score interpretations are both valid and fair (see Chapter 3, American Educational Research Association et al., 2014). When test forms change or when different groups take different test forms, ensuring fair and comparable scores becomes a significant statistical challenge.

To address this challenge, traditional methods for adjusting scores often rely on including common items in the tests - known as anchor items - or assuming that the groups being compared are similar in their distributions of the latent ability the assessment test is designed to measure. These methods aim to adjust for variations in test difficulty and differences in group abilities. However, when no common items are available, these methods may not fully account for group differences, potentially having significant impacts on, for example, academic admission decisions. Complicating matters further, the latent trait levels of the test-takers are not directly observable, which makes it non-trivial to condition the analysis on their values.

In light of these challenges, test score equating has emerged as a routine statistical process for most large-scale testing programs around the world. Test equating methods, used to align scores from different test forms onto a common scale, account for variations in test difficulty and differences in the ability levels of test-taking groups (González & Wiberg, 2017). The choice of equating method depends on assumptions about the test-takers and the available data. When groups of test takers receiving different test forms can be assumed to be similar in their distributions of the ability the test is designed to measure, the Equivalent Groups (EG) design can be used. However, if these groups cannot be assumed equivalent but have completed a set of common items (an anchor test), the nonequivalent groups with anchor test (NEAT) design is suitable (von Davier et al., 2004). When test-taking groups are not similar and no anchor test is administered, but information about test takers’ covariates is available, the nonequivalent groups with covariates (NEC) design can be employed (Wiberg & Bränberg, 2015). Examples of tests with non-equivalent groups but without anchor items include the Invalsi test (INVALSI, 2013), the Armed Services Vocational Aptitude Battery (Quenette et al., 2006), and, until 2011, the Swedish Scholastic Aptitude Test (SweSAT; Stage & Ögren, 2004).

The importance of flexible equating methods became even more apparent during the global spread of Covid-19, which created unprecedented challenges for many large-scale assessments. For instance, the SweSAT faced restrictions on test-taker eligibility, resulting in new demographics taking the test (Wiberg et al., 2021). Despite these changes in the test-taking population, the need to compare scores with previous administrations remained crucial, given the role of SweSAT in college admissions. Historically, researchers addressing changing background distributions of test groups have employed either the NEAT design when an anchor test was available, or the NEC design. However, only a few attempts have been made to integrate information from both covariates and anchor tests, highlighting a gap in the current methodology. Notable exceptions include Wiberg and Bränberg (2015), who explored a case of merging NEAT and NEC designs using categorical covariates and anchor test scores, and Albano and Wiberg (2019), who examined traditional equating methods combining anchor scores with a single covariate. Further, Lu and Guo (2018) used simulations to include information from an anchor test with pseudo equivalent groups (PEG) in a NEAT design. They concluded that if the ability group difference were large, to use only NEAT design was to be preferred over PEG, but the NEAT linking could be improved by using PEG procedures based on background variables. If group ability differences were small, PEG linking produced comparable results to NEAT. Further, Lu and Kim (2021) used statistical matching for equating samples in a NEAT design, while Kim and Walker (2021) used PEG when examining nonequivalent groups caused by suboptimal randomization, a short anchor test of only five items, and minimal collateral information. Recently, Kim and Walker (2022) extended this study when building on the PEG approach and used resampling to evaluate the linking accuracy of group adjustment using sample weights via minimum discriminant information adjustment (MDIA) using test takers’ demographic information, a three-item anchor test, and a mixture of both. They concluded that using both sample weights via MDIA and a short anchor produced the most accurate equating results. More recently, Ozsoy and Kilmen (2023) compared NEAT and NEC designs in a modern equating framework, however they did not combine the two designs.

A promising approach to incorporating information about test takers from covariates is to use propensity scores. Define for each test taker the propensity score e(D) , which is the probability of being assigned a specific treatment (in this case test form) given the covariate vector D (Rosenbaum & Rubin, 1983). Set a treatment variable *Z* equal to 1 if test form Y (active treatment) is administered, and equal to 0 if test form X (control treatment) is administered. Then, the propensity score is defined as e(D)=Pr(Z=1|D) . If D contains every confounder of the relationship between (*X, Y*) and *Z*, the propensity score is a balancing score and it is enough to control for e(D) to create balance in the test groups. The first study to consider propensity scores in test equating was Livingston et al. (1990), who used them for sample matching. This approach was further developed by Yu et al. (2004) and Paek et al. (2006). Subsequent researchers expanded these proposals, with Sungworn (2009) and Powers (2010) using propensity scores to improve traditional equating methods. Moses et al. (2010) took a different approach, using propensity scores to combine two anchor test scores rather than incorporating external covariates in the analysis. Longford (2015) proposed equating based on matching with either inverse proportional weighting or matched pairs, derived from propensity scores based on background variables, while Haberman (2015) employed propensity scores to create PEG from nonequivalent groups before conducting equating.

Wallin and Wiberg (2019) proposed to use propensity scores with the NEAT design, framed within a modern equating framework building on kernel smoothing techniques. Their work demonstrated that stratifying on the propensity scores, an idea dating back to Rosenbaum and Rubin (1984), could achieve a similar level of precision and accuracy compared to the NEAT design, provided the propensity scores are known. Recognizing that propensity scores are never truly known in practice, Wallin and Wiberg (2023) conducted a sensitivity analysis of equated scores to various misspecifications in the propensity score model. Their findings revealed that omitting an important covariate leads to biased estimates of the equated scores, while misspecifying a nonlinear relationship between covariates and test scores increases the equating standard error in the tails of the score distributions. Encouragingly, they also found that the equating estimators are robust against omitting a second-order term and using an incorrect link function in the propensity score estimation model.

Building upon this rich body of research, our paper introduces a novel approach in test equating by combining propensity scores with anchor test scores within the generalized kernel equating framework (Wiberg et al., 2025). An important reason to use kernel equating here is that kernel equating methods are used in practice to equate the college admissions test which we use in the empirical study. While recent studies have utilized propensity scores in kernel equating, none have explored the integration of both propensity scores and anchor test scores in this context, as proposed here. Our overall aim is to examine kernel equating with binary scored items when using propensity scores together with anchor test scores and covariates, comparing this approach with using either only anchor scores in the NEAT design or only propensity scores with the NEC design. We conduct both an empirical study and a simulation study. This allows us to assess the practical implications of our method in a real-world context while also investigating the bias, root mean squared error, and standard errors under varying conditions.

The rest of this paper is structured as follows. In the next section, kernel equating in general is described, followed by a description of kernel equating with propensity scores. This is followed by an empirical study with some results and a simulation study. The last section contains a discussion with some concluding remarks and practical implications.

## Kernel Equating

Kernel equating (von Davier et al., 2004; Wiberg et al., 2025; Wiberg & González, 2016) aims to equate test score *X* to test score *Y* on a target population *T*. For the NEAT and the NEC design, the target population *T* is not trivial to define since we are dealing with samples from two distinct population, *P* and *Q*. It is common to define a synthetic target population, defined symbolically as T=wP+(1−w)Q , with 0≤w≤1. In practice, w>0 is typically used to ensure comparability across administrations.

Kernel equating comprises five steps: (1) Presmoothing, (2) Estimation of the score probabilities, (3) Continuization, (4) Equating, and (5) Evaluating the equating transformation. Denote the observations of *X* and *Y* by $x_{j}$ , j=1,…,J , and $y_{k}$ , k=1,…,K , respectively. Let $r_{j}=\mathrm{Pr}\left(X=x_{j}|T\right)$ and $s_{k}=\mathrm{Pr}\left(Y=y_{k}|T\right)$ be the probabilities of a randomly selected test-taker in the target population *T* scoring $x_{j}$ on test form X and $y_{k}$ on test form Y, respectively. In the first presmoothing step, a log-linear model is typically fitted to the data to reduce the sampling variance. For the NEAT design, denote the observations of anchor test *A* by $a_{l}$ , l=1,…,L , and define the joint probability as $p_{jl}=\mathrm{Pr}\left(X=x_{j},A=a_{l}\right)$ , then

(1) $$\log\left(p_{jl}\right)=\beta_{0}+\overset{T_{r}}{\underset{i=1}{\sum }}\beta_{x,i}x_{j}^{i}+\overset{T_{a}}{\underset{k=1}{\sum }}\beta_{x,i}x_{j}^{i}+\overset{T_{xa}}{\underset{d}{\sum }}\overset{T_{ax}}{\underset{d^{\prime }}{\sum }}\beta_{xa,dd^{\prime }}x_{j}^{d}a_{l}^{d^{\prime }}$$

By estimating the parameters using maximum likelihood estimation, the sample moments are preserved in the distribution being modelled. Several models are typically fitted and the best fitting model according to some criteria is chosen. From the fitted model we obtain the estimated test score probabilities in step 2. If some other proxy of ability is available, such as a propensity score, these can also be modelled in the presmoothing model which will be demonstrated later in the paper.

To obtain the equating transformation, which maps the test scores onto a common scale, we define the cumulative distribution functions (CDFs) of *X* and *Y* in *T* as F(x)=Pr(X≤x|T) and G(y)=Pr(Y≤y|T) , respectively. Kernel equating defines equivalent scores as those that share the same relative position in their respective distributions, using the equipercentile equating transformation:

(2) $$y=\varphi_{Y}\left(x\right)={G_{Y}}^{-1}\left(F_{X}\left(x\right)\right).$$

The equipercentile transformation is the most commonly used method to equate test scores among large-scale testing organizations. As test scores are discrete, continuous approximations of the test score distributions are typically utilized. Kernel equating utilizes kernel functions for this purpose, most commonly a Gaussian kernel function. Let Φ(·) represent the standard normal distribution function, and $r={\left(r_{1},\ldots ,r_{J}\right)}^{t}$ , then the continuized CDF for score *X* is defined as

$$F_{h_{X}}\left(x;r\right)=\mathrm{Pr}\left(X\left(h_{X}\right)\leq x\right)=\underset{j}{\sum }r_{j}\Phi \left(\frac{x-a_{X}x_{j}-\left(1-a_{X}\right)\mu_{X}}{a_{X}h_{X}}\right),$$

where, $\mu_{X}=\underset{j}{\sum }x_{j}r_{j}$ is the mean of *X* in population *T*, $a_{X}=\sqrt{\sigma_{X}^{2}/\left(\sigma_{X}^{2}+h_{X}^{2}\right)}$ , $\sigma_{X}^{2}$ is the variance of *X* in population *T*, and $h_{X}>0$ is the bandwidth which determines the smoothness level of the continuous approximation. The bandwidth can be selected in several ways, and for a comparison of different bandwidth selection methods see Wallin et al. (2021). The continuization of the *Y* score distribution to obtain $G_{h_{Y}}\left(y;s\right)$ , $s={\left(s_{1},\ldots ,s_{K}\right)}^{t}$ , is done in an analogous way. Equation (2) is then used to carry out the equating with these continuized CDFs.

(3) $${\hat{\varphi }}_{Y}\left(x\right)=G_{h_{Y}}^{-1}\left(F_{h_{X}}\left(x\right)\right)$$

Finally, the equating transformation can be evaluated with different measures, including the asymptotic standard error of equating (SEE; von Davier et al., 2004), which, using the delta method, is defined as

(4) $$\text{SEE}\left(x\right)=\sqrt{\text{Var}\left({\hat{\varphi }}_{Y}\left(x\right)\right)}=\|J_{\varphi_{Y}}J_{\text{DF}}C\|.$$

The term ${\hat{\varphi }}_{Y}\left(x\right)$ is defined in equation (3), $J_{\varphi_{Y}}$ represents the Jacobian matrix of the equating function, $J_{\text{DF}}$ denotes the Jacobian matrix of the design function, and **C** is defined such that $\text{cov}\left(v\left(P\right),v\left(Q\right)\right)=CC^{\prime }$ , where $P={\left\{p_{jl}\right\}}_{J\times L}$ and $Q={\left\{q_{kl}\right\}}_{K\times L}$ and v(·) denotes the vectorization of a matrix, where the columns are stacked on top of each other. The design function is defined such that ${\left(r,s\right)}^{\prime }=DF\left(P,Q\right)$ and is, as the name suggests, design specific. See Wallin and Wiberg (2019) and von Davier et al. (2004) for the specific function specification for the NEAT design and NEC design with propensity scores. Lastly, note that the SEE definition gives us a standard error value for each test score *x*. The SEE therefore typically reflects the naturally occurring sparsity of data in the tails of the score distributions (only very few test-takers get a score of 0 or close to 0, and likewise for the highest scores).

## Kernel Equating with the NEAT Design and Categorized Covariates in the NEC Design

To perform kernel equating in the NEAT design we have two choices. First, we can utilize the mixture definition of the target population *T* to construct distributions of *X* and *Y* in *T* and obtain test score probabilities:

(5) $$r_{j}=\mathrm{Pr}\left(X=x_{j}|T\right)=wr_{Pj}+\left(1-w\right)r_{Qj},$$

and

(6) $$s_{k}=\mathrm{Pr}\left(Y=y_{k}|T\right)=ws_{Pk}+\left(1-w\right)s_{Qk},$$

where $r_{Pj}=\mathrm{Pr}\left(X=x_{j}|P\right)$ , $r_{Qj}=\mathrm{Pr}\left(X=x_{j}|Q\right),$ $s_{Pk}=\mathrm{Pr}\left(Y=y_{k}|P\right)$ and $s_{Qk}=\mathrm{Pr}\left(Y=y_{k}|Q\right)$ are the score probabilities of *X* and *Y* in populations *P* and *Q*, respectively. We can then equate the obtained distributions using kernel poststratification equating (KPSE) using equation (3) directly. Secondly, we can link the different test forms through a chain and thus obtain kernel chained equating (KCE), defined as

(7) $$\varphi_{Y}\left(x\right)=G_{h_{Y}}^{-1}\left(H_{h_{Y}}\left(H_{h_{X}}^{-1}\left(F_{h_{X}}\left(x\right)\right)\right)\right)$$

where $H_{h_{Y}}\;\text{and}\;H_{h_{X}}$ are the continuized CDFs for the anchor test forms given to the group that received test form X and test form Y.

If we are using categorized covariates, as in Wiberg and Bränberg (2015), we just exchange the anchor test scores in equations (3) and (7) to the categorized covariate information. How to proceed if we instead of categorized covariates use propensity scores is described next.

## Kernel Equating with Propensity Scores

Wallin and Wiberg (2019) proposed the use of propensity scores in the NEC design with both KPSE and KCE estimators and further expanded the theory in Wallin and Wiberg (2023). One advantage of using propensity scores in test equating is that they summarize multiple covariates into a single scalar, thereby reducing the dimensionality of the problem. This is particularly important when incorporating background variables in log-linear smoothing models, as modelling each covariate directly can lead to sparsity issues - many combinations of test scores and covariate values may have few or no observations, making parameter estimation unstable. By using propensity scores, we avoid sparsity issues while still adjusting for observed confounders.

A fundamental property of propensity scores is that if D contains all confounders of the relationship between test form assignment and test scores then conditioning on e(D) is sufficient to balance the groups. Specifically, we assume that:

$$P\left(X=x_{j}|Z=1,A,e\left(D\right)\right)=P\left(X=x_{j}|Z=0,A,e\left(D\right)\right),$$

which implies that once we control for the anchor score A and the propensity score e(D) , any remaining differences between the groups are random rather than systematic. This balancing assumption allows us to compare test scores fairly between groups, even when direct matching on all covariates is not feasible.

There are multiple ways to estimate propensity scores. In this paper, we use logistic regression, following the common approach of subdividing test takers into strata based on the percentiles of their estimated propensity scores (Rosenbaum & Rubin, 1984). Within each stratum, test takers are assumed to be comparable in ability. The number of strata is chosen based on the covariate distribution to ensure adequate balancing while maintaining a sufficient number of observations in each stratum.

Other methods for balancing covariates include weighting techniques, such as the minimum-variance balancing method proposed by Zubizarreta (2015), which adjusts the empirical distribution of covariates to achieve a prespecified level of balance. Additionally, a range of quantitative and qualitative diagnostics can be used to assess balance between test forms after weighting or stratification. For a comprehensive review of propensity score methods, we refer to Austin and Stuart (2015).

## The KPSE Estimator with Propensity Scores

To obtain a KPSE estimator with propensity scores, i.e., the PS-KPSE estimator, denote the stratified propensity score for strata *l*, l=1,...,L , by $e_{Xl}\left(D\right)$ and $e_{Yl}\left(D\right)$ for populations *P* and *Q*, respectively. Let **d** represent the observed value of **D** and let $p_{jl}=\mathrm{Pr}\left(X=x_{j},e\left(D_{Xl}\right)=e\left(d_{Xl}\right)|P\right)$ and $q_{kl}=\mathrm{Pr}\left(Y=y_{k},e\left(D_{Yl}\right)=e\left(d_{Yl}\right)|Q\right)$ denote the joint probabilities of the test scores and the categorized propensity scores for population *P* and *Q*, respectively. $r_{Qj}$ and $s_{Qk}$ can be estimated directly through ${\hat{r}}_{Pj}=\underset{l}{\sum }{\hat{p}}_{jl}$ and ${\hat{s}}_{Qk}=\underset{l}{\sum }{\hat{q}}_{kl}$ . By design, there is no data to estimate $r_{Qj}$ and $s_{Pk}$ but if we assume that the conditional distributions of *X* given e(D) and *Y* given e(D) is the same in population *P* and *Q* respectively they can be estimated as follows

(8) $${\hat{r}}_{Qj}=\underset{l}{\sum }\left(\frac{{\hat{p}}_{jl}}{\underset{j}{\sum }{\hat{p}}_{jl}}\cdot \underset{k}{\sum }{\hat{q}}_{kl}\right)\text{and}\;{\hat{s}}_{Pk}=\underset{l}{\sum }\left(\frac{{\hat{q}}_{kl}}{\underset{k}{\sum }{\hat{q}}_{kl}}\cdot \underset{j}{\sum }{\hat{p}}_{jl}\right).$$

Equation (8) are then plugged into equations (3), (5), and (6) and we can obtain the PS-KPSE estimator as follows

(9) $$\varphi_{Y}{\left(x;\hat{r},\hat{s}\right)}_{\text{PSE}}=G_{h_{Y}}^{-1}\left(F_{h_{X}}\left(x;\hat{r}\right);\hat{s}\right).$$

### The CE Estimator with Propensity Scores

To define an estimator when using CE with propensity scores, i.e. the PS-KCE estimator, define the continuized CDFs for *X* and *Y* in population *Q* as $F_{h_{P}}\left(x;{\hat{r}}_{P}\right)={\hat{F}}_{h_{P}}\left(x\right)$ , and $G_{h_{Q}}\left(y;{\hat{s}}_{Q}\right)={\hat{G}}_{h_{Q}}\left(y\right)$ , where $r_{P}={\left(r_{P1},...,r_{PJ}\right)}^{t}$ and $s_{Q}={\left(s_{Q1},...,s_{QK}\right)}^{t}$ . However, we also need to define the continuized CDFs *H* for the anchor tests $H_{h_{e_{Xl}}}\left(e_{Xl}\left(d\right);{\hat{t}}_{P}\right)={\hat{H}}_{h_{eXl}}\left(e_{Xl}\left(d\right)\right)$ , and $H_{h_{e_{Yl}}}\left(e_{Yl}\left(d\right);{\hat{s}}_{Q}\right)={\hat{H}}_{h_{eYl}}\left(e_{Yl}\left(d\right)\right)$ , with score probabilities $t_{P}={\left(t_{P1},\ldots ,t_{PL}\right)}^{t}$ and $t_{Q}={\left(t_{Q1},...,t_{QL}\right)}^{t}$ , where $t_{Pl}=\mathrm{Pr}\left(e_{Xl}\left(D\right)=e_{Xl}\left(d\right)|P\right)$ and $t_{Ql}=\mathrm{Pr}\left(e_{Yl}\left(D\right)=e_{Yl}\left(d\right)|Q\right)$ . The PS-KCE estimator can then be defined as

(10) $${\hat{\varphi }}_{Y\left(CE\right)}\left(x\right)=\varphi_{Y\left(CE\right)}\left(x;{\hat{r}}_{P},{\hat{t}}_{P},{\hat{t}}_{Q},{\hat{s}}_{Q}\right)={\hat{G}}_{h_{YQ}}^{-1}\left({\hat{H}}_{h_{e_{Yl}}}\left({\hat{H}}_{h_{e_{Xl}}}^{-1}\left({\hat{F}}_{h_{XP}}\left(x\right)\right)\right)\right).$$

### Combining Anchor Test and Covariate Information

Anchor test information can be incorporated in at least two different ways when using kernel equating with propensity scores. Either they can be incorporated directly in the propensity score model (labelled PSwA) or they can be a separate part of the presmoothing models (labelled PSwoA). If the anchor scores are incorporated directly into the propensity-score model, the resulting presmoothing log-linear models, which we call “inner models”, are obtained:

$$\log\left(p_{jl}\right)=\beta_{0}+\overset{T_{r}}{\underset{i=1}{\sum }}\beta_{x,i}{\left(x_{j}\right)}^{i}+\overset{T_{e}}{\underset{k=1}{\sum }}\beta_{e,k}{\left(e_{l}\right)}^{k}+\overset{T_{xe}}{\underset{d}{\sum }}\overset{T_{ex}}{\underset{d^{\prime }}{\sum }}+\beta_{xe,dd^{\prime }}{\left(x_{j}\right)}^{d}{\left(e_{l}\right)}^{d^{\prime }}$$

If the anchor scores instead are incorporated separately in the log-linear models, we obtain outer models:

$$\log P\left(X=x_{j},e\left(D_{Xl^{\prime }}\right)=e\left(d_{Xl^{\prime }}\right),A=a_{l}\right)=\log\left(p_{jll^{\prime }}\right)=\beta_{0}+\overset{T_{r}}{\underset{i=1}{\sum }}\beta_{x,i}{\left(x_{j}\right)}^{i}+\overset{T_{a}}{\underset{k=1}{\sum }}\beta_{a,k}{\left(a_{l}\right)}^{k}+\overset{T_{e}}{\underset{k=1}{\sum }}\beta_{e,k}{\left(e_{l^{\prime }}\right)}^{k}+\overset{T_{xe}}{\underset{d}{\sum }}\overset{T_{ex}}{\underset{d^{\prime }}{\sum }}\beta_{xe,dd^{\prime }}{\left(x_{j}\right)}^{d}{\left(e_{l^{\prime }}\right)}^{d^{\prime }}+\overset{T_{ea}}{\underset{c}{\sum }}\overset{T_{ae}}{\underset{c^{\prime }}{\sum }}\beta_{ea,cc^{\prime }}{\left(e_{l^{\prime }}\right)}^{c}a_{l}^{c^{\prime }}+\overset{T_{xa}}{\underset{i=1}{\sum }}\overset{T_{ax}}{\underset{k=1}{\sum }}\beta_{xa,dd^{\prime }}x_{j}^{d}a_{l}^{d^{\prime }}.$$

The obtained models are then used to estimate the score probabilities when performing kernel equating.

An alternative approach to presmoothing is the EM-based log-linear method proposed in Liou (1998), which integrates test scores, anchor items, and group membership into a unified model. This approach explicitly accounts for ignorable and nonignorable missing-data mechanisms. While our method shares a conceptual foundation with this framework, it differs in that we use the propensity score e(D) as a scalar balancing measure rather than modelling group membership effects directly. This allows for a flexible incorporation of background covariates while maintaining the benefits of log-linear smoothing

## Empirical Study

Data from the college admissions test SweSAT was used to illustrate the proposed extension of using propensity scores together with information from anchor tests within the kernel equating framework. SweSAT contains 160 multiple-choice, binary scored items, comprising a verbal section and a quantitative section of 80 items each. The two sections are equated separately. The test takers were also administered either an external 40 items (verbal or quantitative) anchor test or 40 (verbal or quantitative) try-out items. Typically, the SweSAT is given twice a year to between 28,000 and 60,000 test takers, and about 2,000 test takers receive the 40 items quantitative anchor test. Prior to introducing the anchor test in the SweSAT, the equating was done by using a set of covariates as described in Lyrén and Hambleton (2011). Although anchor tests are available nowadays, covariates are still of interest when equating the SweSAT as the empirical covariate distributions are not necessary the same at different administrations. Note that currently, when equating the SweSAT, several equating methods are used in practice including the KCE, KPSE and PS equating methods.

We used four administrations of the quantitative section as well as the quantitative anchor test to present two scenarios. For each scenario, we used the same covariates that are recorded and used in past administrations (Altintas & Wallin, 2021; Bränberg et al., 1990; Wallin & Wiberg, 2019, 2023) and the fact that we had access to them. Descriptive statistics of the verbal test scores (range 0–80), age, highest attained education and sex are given in Tables 1 and 2. The verbal SweSAT test scores were grouped into four strata based on previous studies and analyses: [0–32], [33–43], [44–55], and [56–80]. Age was grouped into four strata: [0–20], [21–24], [25–29], [30–oldest], which is like Wallin and Wiberg (2019, 2023) except that we merged the two age categories with few test takers into a single highest age range (30-39 and 40-oldest). Highest attained education (Educ) was grouped into six strata which is reasonable from the Swedish school system: [9y; 9 school years], [AE; Adult education], [G2; 2 years upper secondary school], [G34: 3-4years upper secondary school, [2yC; 2 years of college], [m2yC; more than 2 years of college]. The quantitative anchor test was also used.

**Table 1.** Descriptive statistics of the four administrations in total and for the anchor test groups

		Sex	Age					Educ						Verbal test scores			
Total																	
Adm	*N*	F	−20	21-24	25-29	30-39	40-	9y	AE	G2	G4	2C	m2C	0-33	34-44	45-55	56-80
Y1	55,072	52	58	23	10	6	2	3	1	3	76	9	5	15,542	14,290	12,605	12,635
X1	28,165	56	45	30	13	9	3	2	2	5	70	12	6	6877	7047	7120	7121
Y2	39,246	53	62	21	9	6	3	2	2	3	79	8	5	8501	10,011	9312	10,522
X2	58,990	52	63	23	7	5	2	2	1	3	81	7	3	15,664	15,403	13,736	12,572
Anchor																	
AY1	1578	53	52	24	13	8	2	3	2	4	74	10	5	500	431	355	292
AX1	1299	55	44	28	15	11	3	2	2	6	75	9	4	284	349	337	329
AY2	1727	53	63	21	9	5	2	1	1	4	81	8	5	335	476	448	468
AX2	2615	50	63	25	6	4	1	1	1	1	82	8	3	667	697	711	540

Adm = Administration, *N* = Number of test takers, F = Female percentage, 9y = 9 school years, AE = Adult Education, G2 = 2 years of upper secondary school, G4 = 3-4 years of upper secondary school, 2C = 2 years of college, m2C = more than 2 years of college. AY1, AX1, AY2, AX2 = The anchor test forms given at the same administration as test forms Y1, X1, Y2 and X2.

**Table 2.** Mean, standard deviation (SD) and correlation of the four administrations used in the two scenarios in the empirical study

	Mean	SD	Correlation				
Scenario 1			Sex	Age	Educ	Verb	A
Y1	43.79	12.54	−0.23	−0.16	0.13	0.57	0.84
X1	41.17	15.48	−0.25	−0.16	0.19	0.55	0.89
AY1	19.55	7.59	−0.24	−0.14	0.17	0.54	-
AX1	21.06	7.62	−0.26	−0.10	0.16	0.53	-
			Correlation				
Scenario 2	Mean	SD	Sex	Age	Educ	Verb	A
Y2	46.27	15.23	−0.22	−0.15	0.15	0.58	0.90
X2	45.05	16.25	−0.23	−0.17	0.11	0.55	0.92
AY2	21.75	7.93	−0.25	−0.10	0.16	0.56	-
AX2	22.75	8.14	−0.24	−0.12	0.12	0.56	-

SD = Standard deviation, Educ = maximum education, Verb = Verbal test scores, A = anchor test scores. AY1, AX1, AY2, AX2 = The anchor test forms given at the same administration as test forms Y1, X1, Y2 and X2. The correlation for the variable Sex is point-biserial correlation and Spearman correlation for Educ and Age.

We assumed that we always equated a new test form X to an old test form Y. In scenario 1, we equated two test forms which had very different empirical distributions with respect to sex, age and education compared with all other administrations (see first two rows of Table 1). The reason was that test X1 was administered during a covid year, and it was equated to a test form given before Covid-19. The SweSAT is highly affected by the Swedish unemployment rate, as more test takers want to apply for university if they lose their jobs. The unemployment was higher during covid than the years before the pandemic. In the second scenario, we equated two administrations which had similar empirical distributions with respect to sex, age, and education (row 3 and 4 in Table 1) and the test forms were not administered during the pandemic. KPSE was used to equate the test forms when propensity scores were used.

In each scenario we compared the following method and designs: (1) NEC design with anchor test within the propensity score model (PSwA), (2) NEC design with propensity scores but anchor outside the propensity score model (PSwoA), (3) propensity scores with a NEC design without anchor information (PS), (4) NEAT design with KCE, (5) NEAT design with KPSE.

Propensity scores were obtained with logistic regression using all covariates including the anchor test in 1), and all covariates excluding the anchor test in (2) and 3). The estimated propensity scores from the fitted model were divided into several strata according to the percentiles. The propensity score models were assessed by checking the covariate balance in the strata using the absolute standardized mean difference (ASMD) in which a difference of less than 0.1 indicate good balance (Austin, 2008). The AMSD is defined as

$$\text{ASMD}=\left|\frac{\mu_{D}^{\left(T\right)}-\mu_{D}^{\left(C\right)}}{\sqrt{\frac{\left[\sigma_{D}^{2\left(T\right)}+\sigma_{D}^{2\left(C\right)}\right]}{2}}}\right|,$$

where $\mu_{D}^{\left(T\right)}$ and $\mu_{D}^{\left(C\right)}$ are the means of test form X (treatment) and test form Y (control) for covariate *D* and $\sigma_{D}^{2\left(T\right)}$ and $\sigma_{D}^{2\left(C\right)}$ are their respective variances. We chose to use the number of strata so that this was achieved for as large fraction of strata as possible for every covariate. In our study, this was achieved with 13 strata. The average ASMD for the used covariates when anchor scores were within the propensity scores ranged from 0.02 (Gender) to 0.20 (Anchor) and when the anchor test scores were outside the propensity scores the range was 0.02 (Educ) to 0.15 (Age).

The Bayesian information criterion (BIC, Schwarz, 1978) was used to choose parametrization of the log-linear models in the presmoothing step as it has been shown to have a high selection accuracy for bivariate smoothing (Moses & Holland, 2010). The following log linear models were chosen for KCE and KPSE: ${\;X}^{3},A,AX,AX^{2}$ . For PS without anchor (PS) and PS with anchor inside (PSwA): $X^{3},ps^{2},psX$ and for PS with anchor outside (PSwoA): $X^{3},ps^{2},A,psX,psA$ . Note that, ${\;X}^{3}$ means that all lower terms are also included in the model, i.e. in this case also ${\;X}^{2}$ and X . The Gaussian kernel was used in the continuization step as that is used when kernel equating methods are used to equate the SweSAT.

Summary statistics including correlation are given in Table 2. Note that some of the covariates are quite similar over the four administrations, however for education it differs substantially. The means differed considerably, and the standard deviations differed a lot in scenario 1.

**Figure 1.**
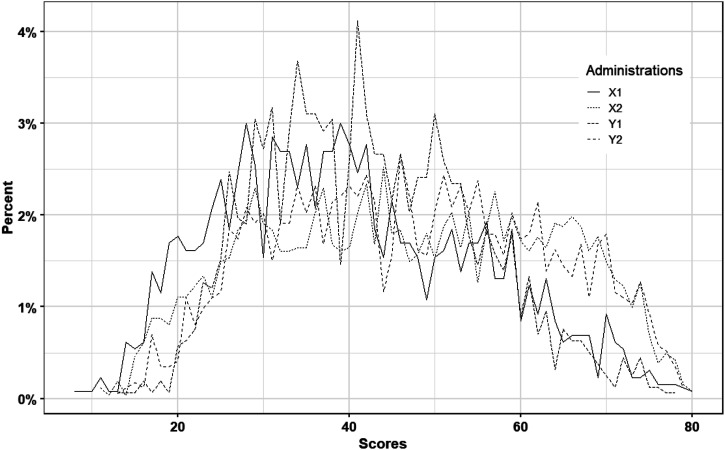
Test Score Distributions for Both Scenario 1 and Scenario 2

Figure 1 displays the four test score distributions, and it is clear from both the mean and SD in Table 2 and Figure 1 that the test distributions are quite different, especially in the mid score range.

To evaluate the equating methods used in the empirical study we used the same measures as Wallin and Wiberg (2019, 2023) used in their empirical studies, i.e., difference between the equated score and the raw score, and the SEE. The empirical study was carried out in R with the package *kequate* (Andersson et al., 2013). To use propensity scores using *kequate*, one can simply replace the function call for the anchor with a call to the estimated and stratified propensity scores.

## Results from the Empirical Study

**Figure 2.**
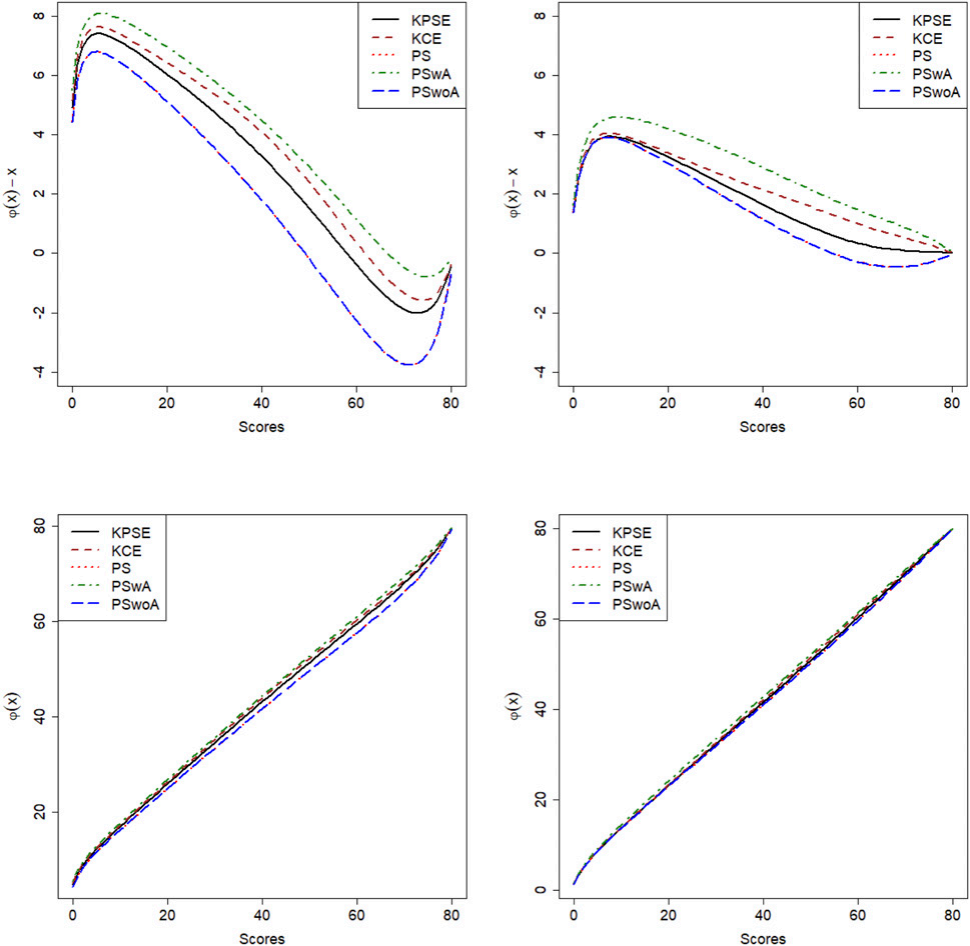
Difference Between Equated Scores and Raw Scores (First Row) and the Equating Transformations (second Row) for Scenario 1 (Left) and Scenario 2 (Right)

The first row in Figure 2 illustrates the difference between equated scores and raw scores and the second row illustrates the equating transformations for the two scenarios when either NEAT design is used (KPSE and KCE) or NEC design with propensity scores is used (PS), or NEC design with anchor test within propensity scores (PSwA) or NEC design with propensity scores but anchor outside (PSwoA). The differences between equated scores and raw scores are much larger for lower test scores and are especially large in scenario 1. Clearly the equating transformations are quite similar, especially in scenario 2 regardless of the method used. In scenario 1, PS and PSwoA differed most from the other equating transformations.

**Figure 3.**
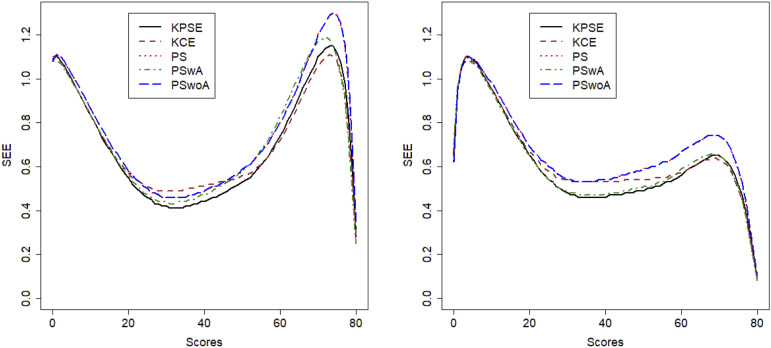
SEE for Scenario 1 to the Left and Scenario 2 to the Right

**Figure A1.**
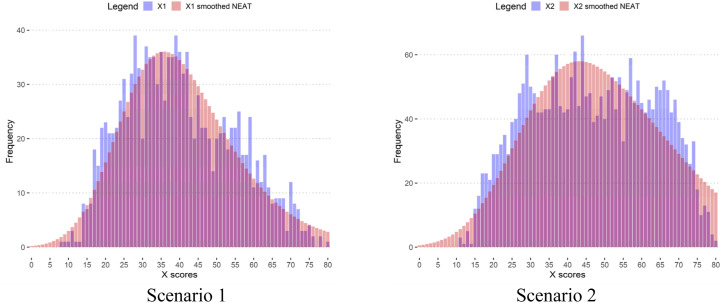
The Comparison Between the Distributions of Non-smoothed and Smoothed With NEAT Model Form X Scores for Scenarios 1 and 2

**Figure A2.**
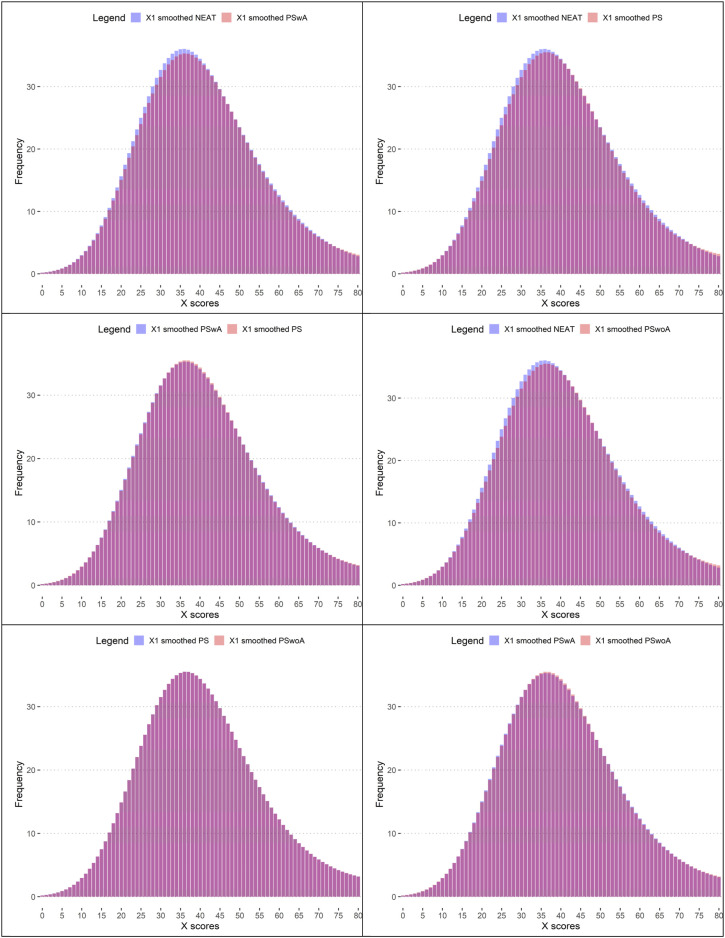
The Comparison Between the Methods for Scenario 1

Figure 3 displays the SEE, and from this figure it is evident that when anchor test scores are included in the propensity scores the SEE is lower than if the anchor test scores are modelled as a separate term in the loglinear presmoothing models. The SEE is much higher in the low and high score range for both scenarios but as expected much lower in scenario 2. It is also interesting to note that SEE for KCE is higher in the mid score range than for the methods using covariate information in both scenarios. To demonstrate how loglinear presmoothing works, we added histograms comparing the distributions of non-smoothed and smoothed Form X scores, as well as comparisons between the methods, and they can be seen in Appendix A, figures A1 and A2.

Summing up, when there are significant differences in the test distributions (Scenario 1), the SEE and the discrepancies between equated scores and raw scores were larger than when the score distributions were more similar (Scenario 2). Also, when anchor test scores are incorporated within the propensity score estimation, we obtained lower SEE compared to when they are treated as separate covariates.

## Simulation Study

To be able to examine several different conditions we conducted a simulation study in which we varied number of test takers, anchor items, and the correlation level between the covariates and the test scores. In addition, the abilities of the test taker groups, and the difficulty of the anchor test were varied. In the following, the simulation design and the evaluation measures re described. For each simulation scenario, 500 replications were used. First, we summarize the scenarios considered, before describing how the simulated data was generated.

• Two populations, P and Q , were generated, each with a population size of 200,000 test takers.
• A subset of either 1,000 or 2,000 test takers was sampled for each replication. A regular test length of 80 and a varying anchor test length of either 20 or 40 were used.
• Low and moderate correlations between the covariates and the test scores were considered.

With two sample sizes, two anchor test lengths, and two correlation scenarios, we had 32 scenarios in total (see Table 3). Next, a description on how the data was generated is given.

**Table 3.** Scenarios (S) in the simulation study

S	P = Q	P	A_b_	A_max_	Weak corr	Moderate corr	*N*
S1	X			20	X		1000
S2	X			20		X	1000
S3	X			40	X		1000
S4	X			40		X	1000
S5		+		20	X		1000
S6		+		20		X	1000
S7		+		40	X		1000
S8		+		40		X	1000
S9	X		+	20	X		1000
S10	X		+	20		X	1000
S11	X		+	40	X		1000
S12	X		+	40		X	1000
S13		+	+	20	X		1000
S14		+	+	20		X	1000
S15		+	+	40	X		1000
S16		+	+	40		X	1000
S17	X			20	X		2000
S18	X			20		X	2000
S19	X			40	X		2000
S20	X			40		X	2000
S21		+		20	X		2000
S22		+		20		X	2000
S23		+		40	X		2000
S24		+		40		X	2000
S25	X		+	20	X		2000
S26	X		+	20		X	2000
S27	X		+	40	X		2000
S28	X		+	40		X	2000
S29		+	+	20	X		2000
S30		+	+	20		X	2000
S31		+	+	40	X		2000
S32		+	+	40		X	2000

*Note.* P = Q: P and Q have similar ability, P = Group P is more (+) capable, A_b_ = Anchor test form is more (+) difficult than the regular test forms, A_max_ = number of anchor items, *N* = sample size.

Note that the simulation condition S23 (or S7 with a smaller sample) is the closest to the empirical study. From the anchor test results presented in Table 2, it is evident that X samples performed better than Y samples. Correlations between the scores and covariates were weak. In operational settings, anchor sample sizes ranged from 1000 to 2000, with the majority being closer to the upper end of that range.

### Data-Generating Process

Two matrices, P and Q , were initialized with dimensions corresponding to the population size ( N ) and the total number of items plus covariates ( $M+L_{1}+L_{2}+L_{3}$ ), where M is the total number of items (regular test items and anchor items) and $L_{1}=3$ , $L_{2}=4$ , and $L_{3}=5$ represent the number of categories for each covariate, respectively. While we used categorized covariates in this study to match our empirical data conditions, the propensity score equating method is flexible and can accommodate continuous covariates as well. Researchers with access to continuous variables such as age or test scores may choose to use them directly in the propensity score estimation without categorization. The choice between categorical and continuous covariates should be guided by data availability and the specific research context.

For each test taker in the population, item responses were generated using the item response theory (e.g. van der Linden, 2018) logistic function:

$$P\left(Y_{ij}=1|\theta_{i}\right)=\frac{1}{1+e^{-a_{j}\left(\theta_{i}-b_{j}\right)}},$$

where $\theta_{i}$ represents the latent ability of test taker i , and $a_{j}$ and $b_{j}$ represents discrimination and difficulty for item *j*, respectively. Latent abilities for populations P and Q were drawn from normal distributions *N* (0,1) and *N* (0.2,1), respectively. The item difficulty parameters were drawn from a *N* (0,1) distribution, and the item discrimination parameters were drawn from a *U* (0.5,2) distribution. All item parameters were drawn independently of each other. The binary responses were then determined by comparing the logistic probability to a uniform random variable:

$$Y_{ij}=\left\{ \begin{array}{l} 1,\;\text{if}\;\frac{1}{1+\exp\left(-a_{j}\left(\theta_{i}-b_{j}\right)\right)}>U\\ 0,\;\text{otherwise} \end{array}\right.$$

where U ∼U(0,1) . The sum scores for the regular test items and the anchor items from the generated item responses were computed for each test taker. The covariates for each test taker were generated similarly. For each test taker i and covariate k ,

$$P\left(C_{k,i}=1|\theta_{i}\right)=\frac{1}{1+e^{-a_{C_{k}}\left(\theta_{i}-b_{C_{k}}\right)}},$$

where $b_{C_{k}}∼N\left(0,1\right)$ and the covariate item parameters $a_{C_{k}}$ were generated according to different correlation structures:

• Low correlation setting: $a_{C_{k}}∼U\left(0.1,0.5\right)$
• Moderate correlation setting: $a_{C_{k}}∼U\left(0.5,1.5\right)$

Lastly, we calculated the sum score of each covariate, thus creating three categorical covariates.

### Population Model and Four Estimators

We examined a population model and four alternative estimators. For each scenario and estimator, the best-fitting log-linear models were selected separately using the Akaike information criterion (AIC; Akaike, 1974), BIC, and the likelihood ratio test (LRT; Haberman, 1974a; 1974b). This resulted in at most six unique models – one for ( X,A ) and one for ( Y,A ) per criterion. In the second step, all possible combinations of these model pairs were evaluated, and the pair that minimized the average SEE across test scores was chosen, following the approach suggested by Wallin and Wiberg (2024).

*Population model:* Using the population-level data, propensity scores were estimated using a logistic regression model with the covariates as predictors. Test takers were stratified into 15 groups based on these propensity scores. Equating was thereafter performed using KPSE and KCE methods.

*Common procedure for Estimators 1-3:* For all propensity score estimators, test takers were stratified into 15 groups based on propensity scores, and the strata acted as predictors in the log-linear model together with the score variables. The estimators differ in their propensity score specification:

Estimator 1: Equating with propensity score that includes only covariates (PS)

• Propensity scores were estimated using covariates only.
• Equating was performed using KPSE and KCE methods.

Estimator 2: Equating with propensity scores that includes both covariates and anchor scores (PSwA)

• Propensity scores were estimated including both covariates and anchor items.
• Equating was performed using KPSE and KCE methods.

Estimator 3: Equating with anchor score outside of propensity score (PSwoA)

• Propensity scores were estimated only with covariates.
• A three-dimensional contingency table was created for the sum score, propensity score strata, and anchor score.
• Equating was performed using the PSE method.

Estimator 4: NEAT Equating (KCE/KPSE)

• Kernel equating with the NEAT design using both KCE and KPSE was conducted as a baseline comparison.

### Evaluation Measures

To evaluate the equating transformations, we used four evaluation measures. We examined bias, over R replications

$$\text{Bias}\left({\hat{\varphi }}_{Y}\left(x_{i}\right)\right)=\frac{1}{R}\overset{R}{\underset{g=1}{\sum }}\left({\hat{\varphi }}_{Y}^{\left(g\right)}\left(x_{i}\right)-\varphi \left(x_{i}\right)\right),$$

where $\varphi \left(x_{i}\right)$ is the true equating transformation. The true equating transformations for each estimator (population-level KPSE and KCE) were defined based on the true propensity scores, which were calculated using a logistic function of the anchor scores and the covariates. The NEAT KCE estimators were compared against the identity function, a valid procedure due to the data-generating process with difficulty parameters drawn from the same distribution (Laukaityte & Wiberg, 2024, Leoncio, Wiberg, & Battauz, 2023). The equating transformations were then derived from log-linear models fit to the population-level frequency tables of test scores and categorized propensity scores. This setup ensured that the equating transformations reflected the true relationship between the test scores, the covariates and the anchor scores.

We examined the SEE from equation (4), and the root mean squared error (RMSE),

$$\text{RMSE}\left({\hat{\varphi }}_{Y}\left(x_{i}\right)\right)=\sqrt{\frac{1}{R}\overset{R}{\underset{g=1}{\sum }}{\left({\hat{\varphi }}_{Y}^{\left(g\right)}\left(x_{i}\right)-\varphi \left(x_{i}\right)\right)}^{2}},$$

and the standard error (SE)

$$\text{SE}\left({\hat{\varphi }}_{Y}\left(x_{i}\right)\right)=\sqrt{\frac{1}{R-1}\overset{R}{\underset{g=1}{\sum }}{\left({\hat{\varphi }}_{Y}^{\left(g\right)}\left(x_{i}\right)-{\bar{\varphi }}_{Y}^{g}\right)}^{2}},$$

where and ${\bar{\varphi }}_{Y}^{g}=\frac{1}{R}\overset{R}{\underset{g=1}{\sum }}\varphi_{Y}^{\left(g\right)}\left(x_{i}\right)$ . The simulation study was carried out in R with the R package *kequate* (Andersson et al., 2013). The used code can be found on the following github: https://github.com/gabrieltwallin/Equating_anchor_PS.

## Results from the Simulation Study

**Figure 4.**
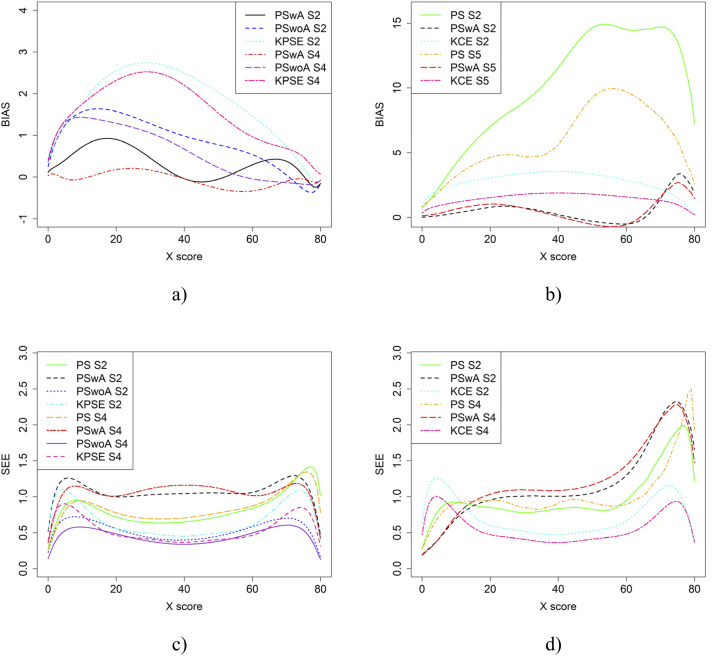
Bias (a and b) and SEE (c and d) for the Baseline Case when Correlation Between Covariates was Moderate Strong and the Size of an Anchor Test Form was Either 20 Items (S2) or 40 items (S4). Note that for KPSE, we do not Show the Bias Results for the PS Method, as the Bias is so Large (see Appendix Figures B1(a) and B2(a)) that it Obscures the Differences Between the other Methods. For KCE, we Used an Identity Function as a Criteria Function when Evaluating Bias for NEAT KCE. Otherwise, it Resulted in Large Bias Values Similar to the PS Results for KPSE (see Appendix Figures B1(a) and B2(a))

**Figure 5.**
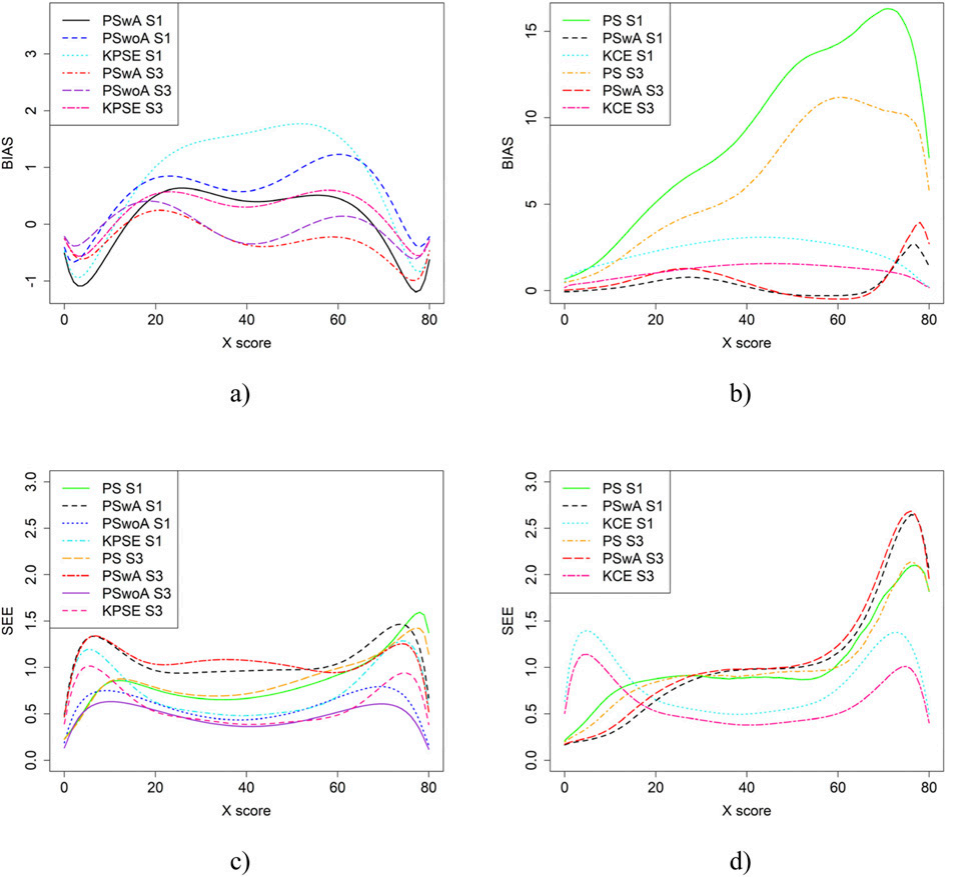
Bias (a and b) and SEE (c and d) for the Baseline Case when Correlation Between Covariates was Weak and the Size of an Anchor Test Form was Either 20 Items (S1) or 40 items (S3)

In the simulation study, in addition to varying sample sizes, anchor test lengths, and correlation strength, we also varied the abilities of the test-taker groups and the difficulty of the anchor test. Note, in all figures in this section the left figures are based on the KPSE estimator, and the right figures are based on the KCE estimator. Figures 4 and 5 present the results for the baseline case where the groups had similar abilities, and the difficulty of the regular test forms and the anchor test form were comparable. The difference between the two figures is the strength of the correlation between the covariates. When the correlation between the covariates was moderately strong (see Figure 4(a) and (b)), the differences in bias between the studied equating methods were larger compared to when the correlation was weak (see Figure 8(a, b)), especially for KPSE. However, the differences in bias between the various anchor test lengths were more pronounced when the correlation was weak. For KCE, the differences in bias across different correlation strengths or anchor test lengths were small.

The main differences in SEE were observed between the different methods, with the smallest SEE occurring when the anchor score was outside of the propensity score (PSwoA) and the anchor test consisted of 40 items for KPSE (see Figures 4(c) and 5(c)), and for the NEAT design when using KCE (see Figures 4(d) and 5(d)).

**Figure 6.**
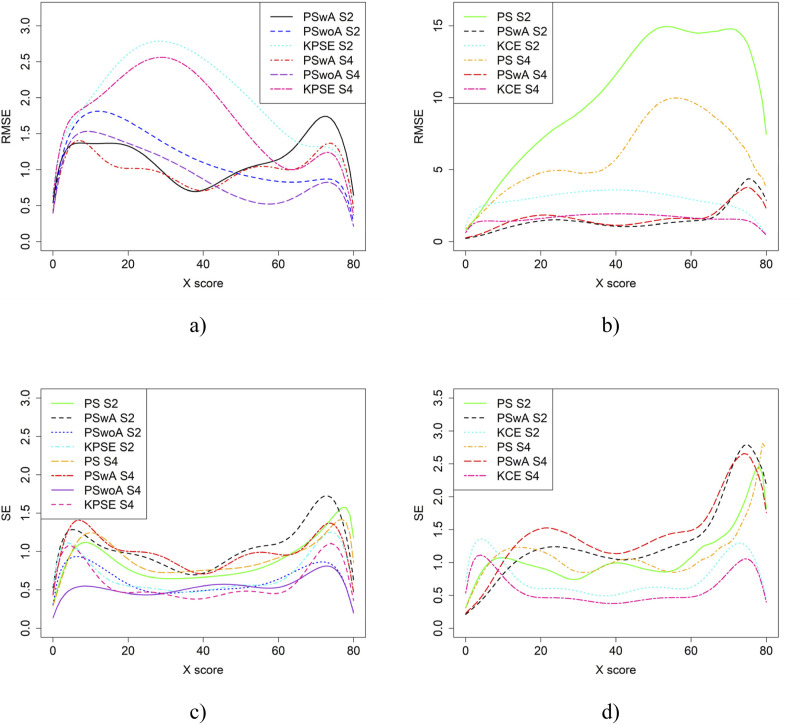
RMSE (a and b) and SE (c and d) for the Baseline Case when Correlation Between Covariates was Moderate and the Size of an Anchor Test Form was Either 20 Items (S2) or 40 items (S4)

Figure 6 displays the RMSE and SE for the baseline case in Figure 4. As their results are similar to the bias and SEE figures – we draw the same conclusions from them. For subsequent scenarios, we have therefore omitted RMSE and SE figures, but these can be obtained upon request from the corresponding author.

**Figure 7.**
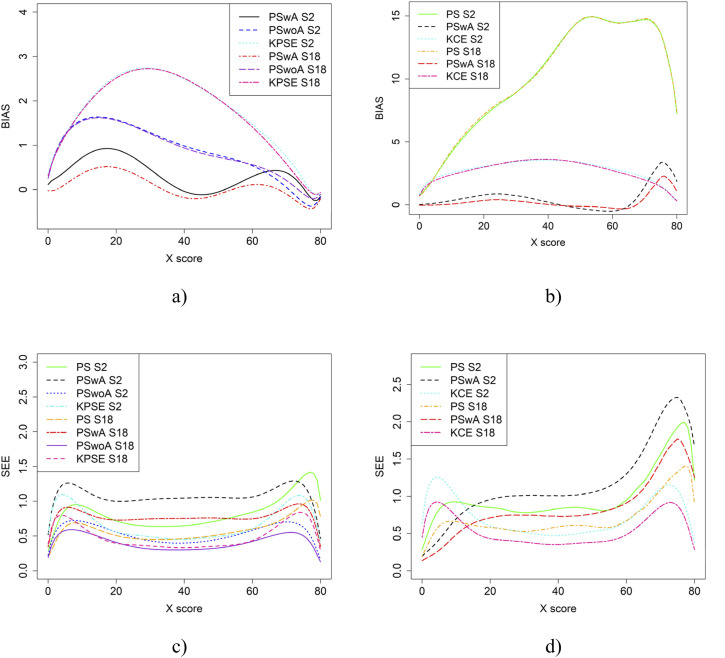
Bias (a and b) and SEE (c and d) for the Baseline Case when Correlation Between Covariates was Moderate and the Sample Size *N* was Either 1000 (S2) or 2000 (S18)

Figure 7 presents the results for bias and SEE for the baseline case for different sample sizes: 1000 (S2) and 2000 (S18). The bias only indicated minimal or no differences. The sample size, however, impacted the SEE results, with the largest differences occurring for equating with propensity scores (PS) and with propensity scores that included both covariates and anchor scores (PSwA).

**Figure 8.**
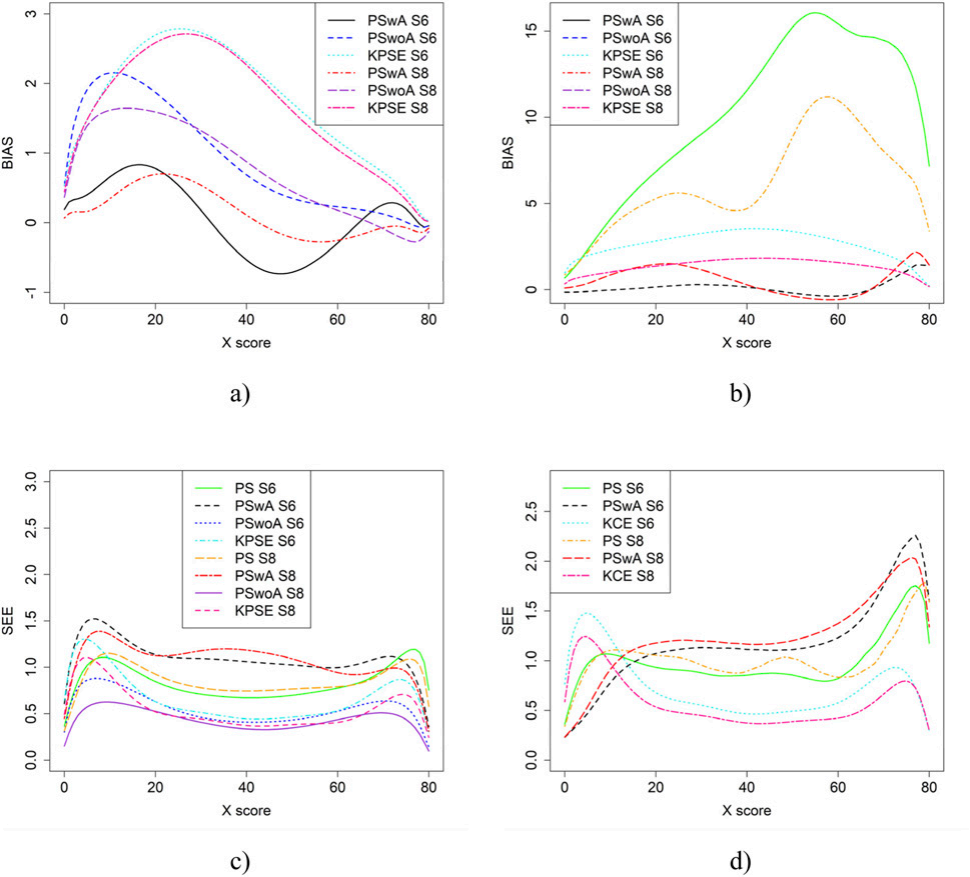
Bias (a and b) and SEE (c and d) for Groups Differing in Ability when Correlation Between Covariates was Moderate and the Size of an Anchor Test Form was Either 20 Items (S6) or 40 items (S8)

**Figure B3.**
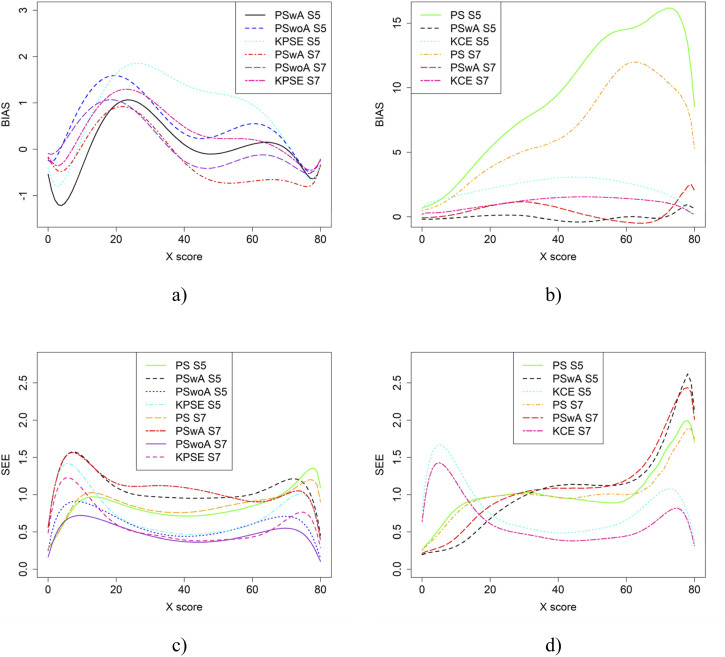
Bias (a and b) and SEE (c and d) for Groups Differing in Ability when Correlation Between Covariates was Weak and the Size of an Anchor Test Form was Either 20 Items (S5) or 40 Items (S7)

Figure 8 displays result similar to those shown in Figure 4. However, in this case, the scenarios involve one group with average ability and another with higher ability. SEE values for KPSE are nearly identical to those in the baseline case shown in Figure 4. For KCE, when the correlation between the covariates is moderate, SEE values are slightly lower at the high scores, especially for PswA and PS with longer anchor test (see Figure 10(b)) compared to the baseline case. In contrast, when correlation is weak, SEE values are higher at the high scores for these same methods (see Appendix Figure B3). The differences in group abilities had a slightly greater impact on bias values, particularly for KPSE compared to KCE. When the correlation between the covariates was weak, bias increased for the lower scores for KPSE equating, unlike in the baseline case.

**Figure 9.**
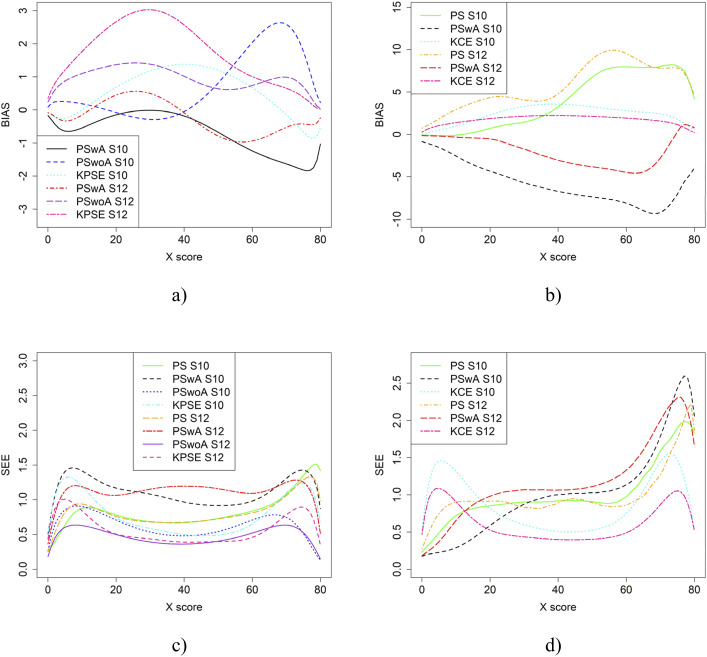
Bias (a and b) and SEE (c and d) for Groups Similar in Ability when Anchor Test Form was More Difficult than the Regular Test Forms, Correlation Between Covariates was Moderate and the Size of an Anchor Test Form was Either 20 Items (S10) or 40 items (S12)

If the anchor test form is more difficult than the regular test forms, the bias results change significantly, especially when the correlation between the covariates is moderate (see Figure 9). The largest changes in bias are observed for equating methods using propensity scores. Interestingly, when the correlation between the covariates was weak, the bias results for KCE were similar to the baseline case (see Figure 4). The difficulty of the anchor test had only a minor effect on SEE values.

**Figure 10.**
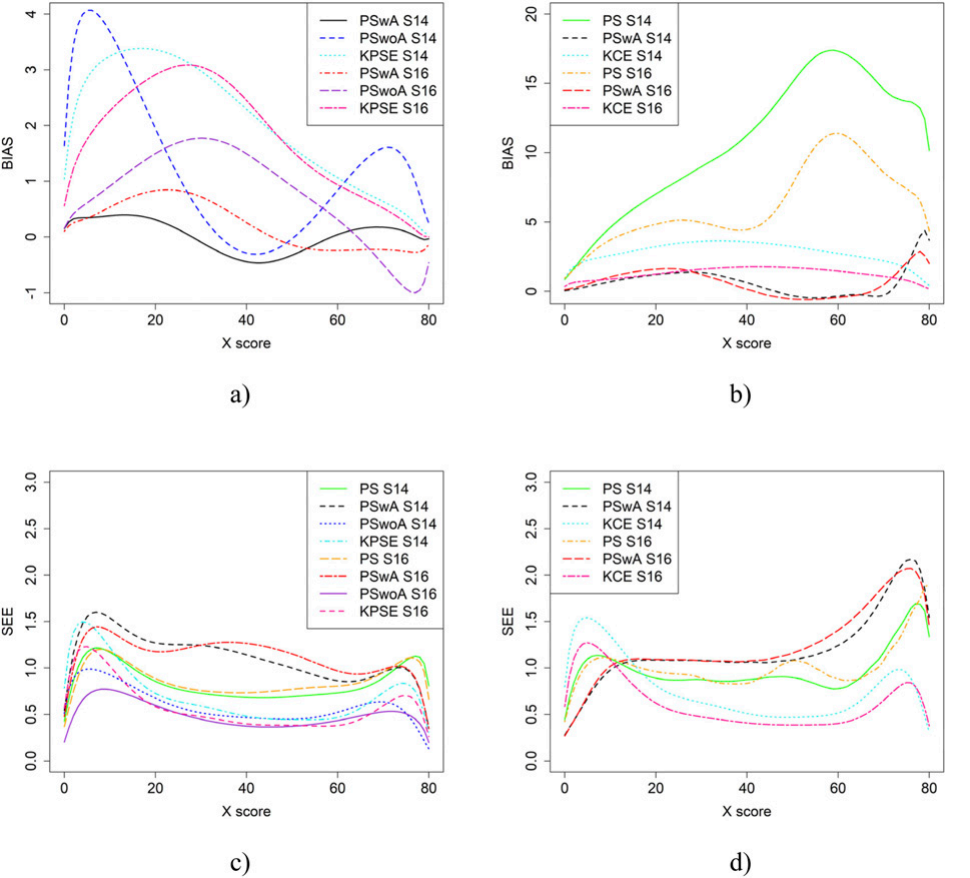
Bias (a and b) and SEE (c and d) for Groups Differing in Ability when Anchor Test Form was More Difficult than the Regular Test Forms, Correlation Between Covariates was Moderate and the Size of an Anchor Test Form was Either 20 Items (S14) or 40 items (S16)

Figure 10 displays the equating results when the anchor test form is more difficult than the regular test forms and one group has higher ability than the other. For the KPSE based methods, PSwoA had the lowest SEE and PSwA had in general the lowest bias. The greatest impact on bias values was seen for the KPSE methods, compared with the KCE methods.

## Discussion

The primary objective of this study was to propose and evaluate a novel approach that could be incorporated into generalized kernel equating. The proposed approach integrates propensity scores with anchor test scores. This approach was compared against two established methods: the NEAT design alone and the NEC design using propensity scores without anchor tlest scores. The empirical study showed that when test distributions exhibited significant differences (Scenario 1), the SEE and the discrepancies between equated scores and raw scores were more pronounced. This finding aligns with the results of Laukaityte and Wiberg (2024). Notably, the use of propensity scores led to a lower SEE, consistent with the conclusions of Wallin and Wiberg (2019), although their study did not examine the combined use of anchor test scores, and propensity scores derived from covariate information. As expected, incorporating anchor test scores within the propensity score estimation resulted in a lower SEE compared to treating them as separate covariates. This suggests that the integration of anchor test information into propensity scores may enhance the precision of equating. This is also in line with the results of Kim and Walker (2021; 2022) who concluded that using both sample weights via MDIA and a short anchor produced the most accurate equating results.

From the simulation study, we concluded that when the correlation between the covariates was moderately strong, the differences in bias and RMSE between the methods were larger compared to when the correlation was weak, especially when using KPSE. The difference was also more pronounced when a shorter anchor test was used in conjunction with weak correlation. This is not surprising, as a shorter anchor test and weaker correlation yield less overall information. For KCE, the differences in bias across different correlation strengths or anchor test lengths were small. For KPSE, the smallest SEE and SE occurred when the anchor score was outside of the propensity score (PSwoA) and the anchor test consisted of 40 items. For the NEAT design, the smallest SEE and SE occurred when using KCE. This is expected, as more information about the test takers should yield a smaller error, as seen, for example, in Bränberg and Wiberg (2011), who examined observed score linear equating with covariates.

Varying the sample sizes had little effect on bias but did impact the SEE results, with the largest differences occurring when equating with propensity scores (PS) and with propensity scores that included both covariates and anchor scores (PSwA). In general, SEE was lower when anchor test scores were used as a separate covariate (PSwoA) compared to when they were included within the propensity score (PSwA). This is probably because treating the anchor scores as a separate covariate provides more information about the test takers than when the anchor scores are combined with other covariates within the propensity score.

When the ability between the groups differed, the SEE values for KPSE were nearly identical to those in the baseline case. This is in line with Lu and Guo (2018) who concluded that if the ability group difference were large, to use NEAT is preferred in terms of RMSE and bias, instead of using only information in background variables through PEG. Also, our conclusion to use background information together with anchor test information is in line with their conclusion of using PEG procedures based on background variables together with the anchor test to improve the equating. When group ability differences were small (baseline case), the SEE were low when either PSwoA or NEAT (KCE/KPSE) were used. Although we used a different approach and used bias and SEE to evaluate, our result is in line with Lu and Guo (2018), who concluded that using only NEAT design compared with using PEG without an anchor test gave comparable results, in terms of bias and RMSE.

When the correlation was moderate, SEE values were slightly lower at the higher scores, especially for PSwA and PS with a longer anchor test when KCE was used. This result is contrary to the findings of Ricker and von Davier (2007), who concluded that a shorter anchor yields a larger bias for KCE compared to KPSE; however, they did not examine the effect of correlation. Note that when the correlation was weak, the SEE values are higher at the higher scores for the same methods. The differences in group abilities had a slightly greater impact on the bias values, especially for KPSE compared to KCE. This is in line with, for example, Puhan (2010) and Powers and Kolen (2014), who concluded that CE is less affected by group differences. When the correlation between the covariates was weak, bias increased for the lower scores for KPSE equating, unlike in the baseline case. Lu and Guo (2018) conclusion that if the anchor test is weak (i.e. few items and low correlation), is like the conclusion here, i.e. that we can then improve the equating with background information. When the anchor test form is more difficult than the regular test form, the bias results change significantly, particularly when the correlation between the covariates is moderate. Notably, the bias is especially large when using propensity scores without anchor test information. A possible explanation is that the propensity scores diverge too much from the anchor test scores, though this requires further investigation.

In summary, when the anchor test form is more difficult than the regular test forms and one group has higher ability than the other, the bias was more affected when using KPSE methods compared to KCE methods. These results are consistent with those of Laukaityte and Wiberg (2024), who studied how differences in group abilities impact kernel equating methods. If one has access to covariates, it is advisable to include them in the presmoothing model, as this can reduce the SEE. When multiple covariates at different levels are available, using propensity scores is an effective way to incorporate a large amount of information. In our study, it was also evident that, in terms of bias and SEE, it is better to include the anchor scores as a standalone covariate rather than incorporating them into the propensity score model.

This study has some limitations. First, we included only binary-scored items; in the future, polytomously scored items and mixed-format tests incorporating information from covariates should be examined. For example, Wallmark et al. (2023) examined kernel equating in mixed-format tests. A second limitation is that we examined only a few presmoothing models. Wallin and Wiberg (2020; 2024) have shown that the presmoothing model has a significant impact on the equating transformation; therefore, several other models should be explored in future research. Another limitation concerns the choice of covariates and future studies should investigate other covariates and their usefulness when equating test scores. Note, there is a trade-off between test takers performance and precision of the test depending on the design of the test. On one hand, to include an anchor test prolongs the testing time and thus makes test takers more fatigue, on the other hand more information about the test takers is collected when including an anchor test and thus the precision of the equating can be increased.

While our primary focus was on horizontal equating scenarios where test-taker ability distributions differ but test content is similar, our approach may also be applicable to vertical equating. In vertical equating, test forms are tailored for different school grades, introducing additional complexities in modelling ability differences. As suggested in prior work (Liou, 1998), nonignorable missing-data models may be more appropriate in such contexts. Our method could potentially be adapted for vertical equating by extending the log-linear model to include additional covariates representing developmental differences across grades. Exploring this extension remains an interesting possibility for future research. Furthermore, continuous propensity scores can be used directly as conditioning variables in equating without requiring stratification, similar to how anchor scores function in traditional equating designs.

Another limitation is that the current standard error estimation approach does not explicitly account for the covariance between the empirical distributions *F* and *G* in the synthetic population. While we follow the framework of Wallin and Wiberg (2019) which provides estimates for the variances within each distribution, incorporating the covariance component would provide more accurate standard error estimates for the equated scores. Future methodological work should address how this covariance can be systematically incorporated into the standard error calculations for propensity score equating methods.

Finally, given that adjusting test score scales is a practical issue in many large-scale assessments, we included an empirical study to address this problem. Our results suggest that utilizing information from covariates, when they are available and informative, can be beneficial. However, we emphasize that an anchor test should also be used if available.

## Figures and Tables

**Figure B1.**
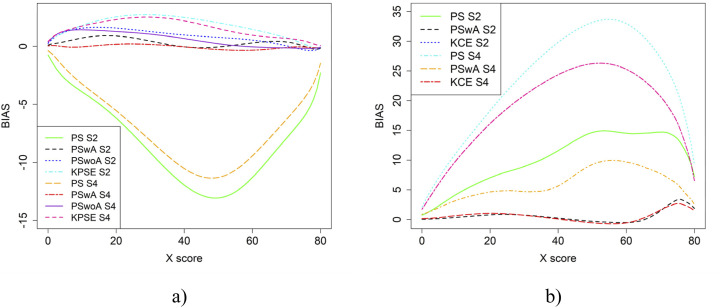
Bias for the Baseline Case when Correlation Between Covariates was Moderate and the Size of an Anchor Test Form was Either 20 Items (S2) or 40 Items (S4)

**Figure B2.**
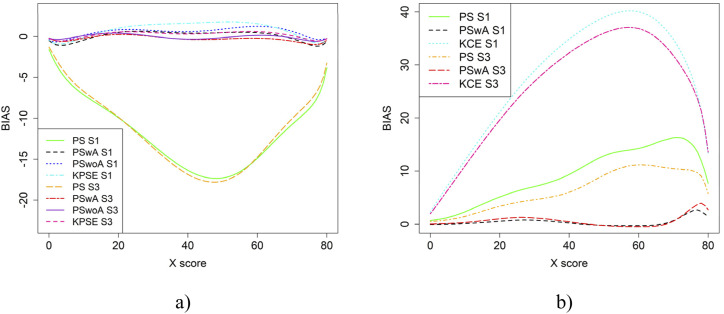
Bias for the Baseline Case when Correlation Between Covariates was Weak and the Size of an Anchor Test Form was Either 20 Items (S1) or 40 Items (S3)

## Appendix A

The comparison between the distributions of non-smoothed and smoothed with NEAT model Form X scores for scenarios 1 and 2 is presented in Figure A1. Smoothing results with other models are almost identical to the presented ones and thus are omitted.

Figure A2 shows the comparison of smoothed distributions between different models used in the study. Only the results for Scenario 1 are presented here, as the results for Scenario 2 are almost identical.
